# Long-Term Use of Statins Lowering the Risk of Rehospitalization Caused by Ischemic Stroke Among Middle-Aged Hyperlipidemic Patients: A Population-Based Study

**DOI:** 10.3389/fphar.2021.741094

**Published:** 2021-10-18

**Authors:** Jiu-Haw Yin, Giia-Sheun Peng, Kang-Hua Chen, Chi-Ming Chu, Wu-Chien Chien, Li-Ting Kao, Chia-Chao Wu, Chih-Wei Yang, Wen-Chiuan Tsai, Wei-Zhi Lin, Yi-Syuan Wu, Hung-Che Lin, Yu-Tien Chang

**Affiliations:** ^1^ Department of Neurology, Tri-Service General Hospital, National Defense Medical Center, Taipei, Taiwan; ^2^ Division of Neurology, Department of Internal Medicine, Taipei Veterans General Hospital, Hsinchu Branch, Hsinchu County, Taipei, Taiwan; ^3^ Associate Professor, School of Nursing, College of Medicine, Chang Gung University, Taoyuan, Taiwan; ^4^ Associate Research Fellow, Department of Nursing, Chang Gung Memorial Hospital, Tao-Yuan Branch, Taoyuan, Taiwan; ^5^ School of Public Health, National Defense Medical Center, Taipei, Taiwan; ^6^ Department of Surgery, Songshan Branch of Tri-Service General Hospital, National Defense Medical Center, Taipei, Taiwan; ^7^ Division of Biostatistics and Informatics, Department of Epidemiology, School of Public Health, National Defense Medical Center, Taipei, Taiwan; ^8^ Department of Public Health, China Medical University, Taichung, Taiwan; ^9^ Department of Healthcare Administration and Medical Informatics College of Health Sciences, Kaohsiung Medical University, Kaohsiung, Taiwan; ^10^ Department of Medical Research, Tri-Service General Hospital, National Defense Medical Center, Taipei, Taiwan; ^11^ Department of Pharmacy Practice, Tri-Service General Hospital, Taipei, Taiwan; ^12^ School of Pharmacy, National Defense Medical Center, Taipei, Taiwan; ^13^ Graduate Institute of Life Sciences, National Defense Medical Center, Taipei, Taiwan; ^14^ Division of Nephrology, Department of Medicine, Tri-Service General Hospital, National Defense Medical Center, Taipei, Taiwan; ^15^ Division of Gastroenterology, Department of Internal Medicine, Tri-Service General Hospital, National Defense Medical Center, Taipei, Taiwan; ^16^ Department of Pathology, Tri-Service General Hospital, National Defense Medical Center, Taipei, Taiwan; ^17^ School of Medicine, National Defense Medical Center, Taipei, Taiwan; ^18^ Graduate Institute of Medical Sciences, National Defense Medical Center, Taipei, Taiwan; ^19^ Department of Otolaryngology-Head and Neck Surgery, Tri-Service General Hospital, National Defense Medical Center, Taipei, Taiwan; ^20^ Hualien Armed Forces General Hospital, Hualien, Taiwan

**Keywords:** statins, lipid-lowering medicines, rehospitalization, hyperlipidemia, ischemic stroke, secondary prevention, diabetes mellitus

## Abstract

**Background:** The long-term effects of statin use on rehospitalization due to ischemic stroke (reHospIS) in hyperlipidemic patients are still unknown. Therefore, we aimed to assess the long-term risks of reHospIS for hyperlipidemic patients who were taking statins and nonstatin lipid-lowering medicines on a regular basis.

**Methods and Materials:** The National Health Insurance Research Database in Taiwan was used to conduct a 6-year cohort study of patients >45 years old (*n* = 9,098) who were newly diagnosed with hyperlipidemia and hospitalized for the first or second time due to ischemic stroke (IS). The risk of reHospIS was assessed using Cox proportional hazards regression model.

**Results:** Nonstatin lipid-lowering medicines regular users were associated with a higher risk of reHospIS compared to stains users (hazard ratio, HR = 1.29–1.39, *p* < 0.05). Rosuvastatin was the most preferred lipid-lowering medicine with lower HRs of reHospIS in hyperlipidemic patients whether they developed diabetes or not. Bezafibrate regular users of hyperlipidemic patients developing diabetes (HR = 2.15, *p* < 0.01) had nearly 50% lower reHospIS risks than those without diabetes (HR = 4.27, *p* < 0.05). Age, gender, drug dosage, comorbidities of diabetes and heart failure (HF), and characteristics of the first hospitalization due to IS were all adjusted in models. Moreover, increasing trends of HRs of reHospIS were observed from Rosuvastatin, nonstatin lipid-lowering medicines, Lovastatin, and Gemfibrozil to Bezafibrate users.

**Conclusion:** Statins were associated with long-term secondary prevention of reHospIS for hyperlipidemic patients. Rosuvastatin seemed to have the best protective effects. On the other hand, Bezafibrate appears to be beneficial for hyperlipidemic patients developing diabetes. Further research into the combination treatment of statin and nonstatin lipid-lowering medicines in hyperlipidemic patients developing diabetes is warranted.

## Introduction

Hyperlipidemia is one of the most prevalent risk factors for atherosclerosis and cardioembolic stroke (Ayata et al., 2013), particularly in patients with high LDL cholesterol (Farnier and Davignon, 1998; MF, 2021). Ischemic stroke (IS) is a major cause of morbidity and mortality. Elevated LDL levels appear to increase the risk of IS (Tziomalos et al., 2009). Treatment of hyperlipidemia is helpful in both primary and secondary prevention of coronary heart disease and stroke (Arshad, 2014).

Statins (3-hydroxy-3-methylglutaryl coenzyme A reductase inhibitors) are the most commonly prescribed lipid-lowering medicines (Bonetti et al., 2003). They have been shown to reduce the risk of IS in patients with a history of IS (Tziomalos et al., 2009) via the lipid-lowering effect (Farnier and Davignon, 1998) and the reduction of platelet activation and reactivity (Pawelczyk et al., 2015). Statin-based lipid lowering is effective for both primary and secondary prevention of IS (Milionis et al., 2020; Zhu et al., 2020). In addition, statin pretreatment or use in the acute phase of IS improved outcomes for recurrence, cardiovascular events (Farnier and Davignon, 1998; Flint et al., 2012a; Kim et al., 2014; O’Brien et al., 2015; Guo et al., 2015; Scheitz et al., 2015; Yeramaneni et al., 2017; Cui et al., 2020; Furlan et al., 2020; Wang et al., 2020), neurological disability, and all-cause and cardiovascular mortality (Orkaby et al., 2020). Statins are the first-line LDL-lowering therapy in diabetic patients. Studies indicated that adding nonstatin lipid-lowering medicines to statins could improve the lipid profile (Scicali et al., 2018) and reduce adverse cardiovascular events (NAEEM et al., 2018) in diabetic individuals.

However, most previous studies (Flint et al., 2012b; Koton et al., 2012) compared the short-term protective effects of statin users, inpatient statin users, or pre-IS stroke statin users to statin-naïve users. To the best of our knowledge, no long-term follow-up studies have been conducted to evaluate the risk of rehospitalization due to ischemic stroke (reHospIS) in hyperlipidemic patients with or without diabetes who were regularly taking statins or other nonstatin lipid-lowering medicines.

## Methods and Materials

### Study Population and Study Design

**FIGURE 1:**
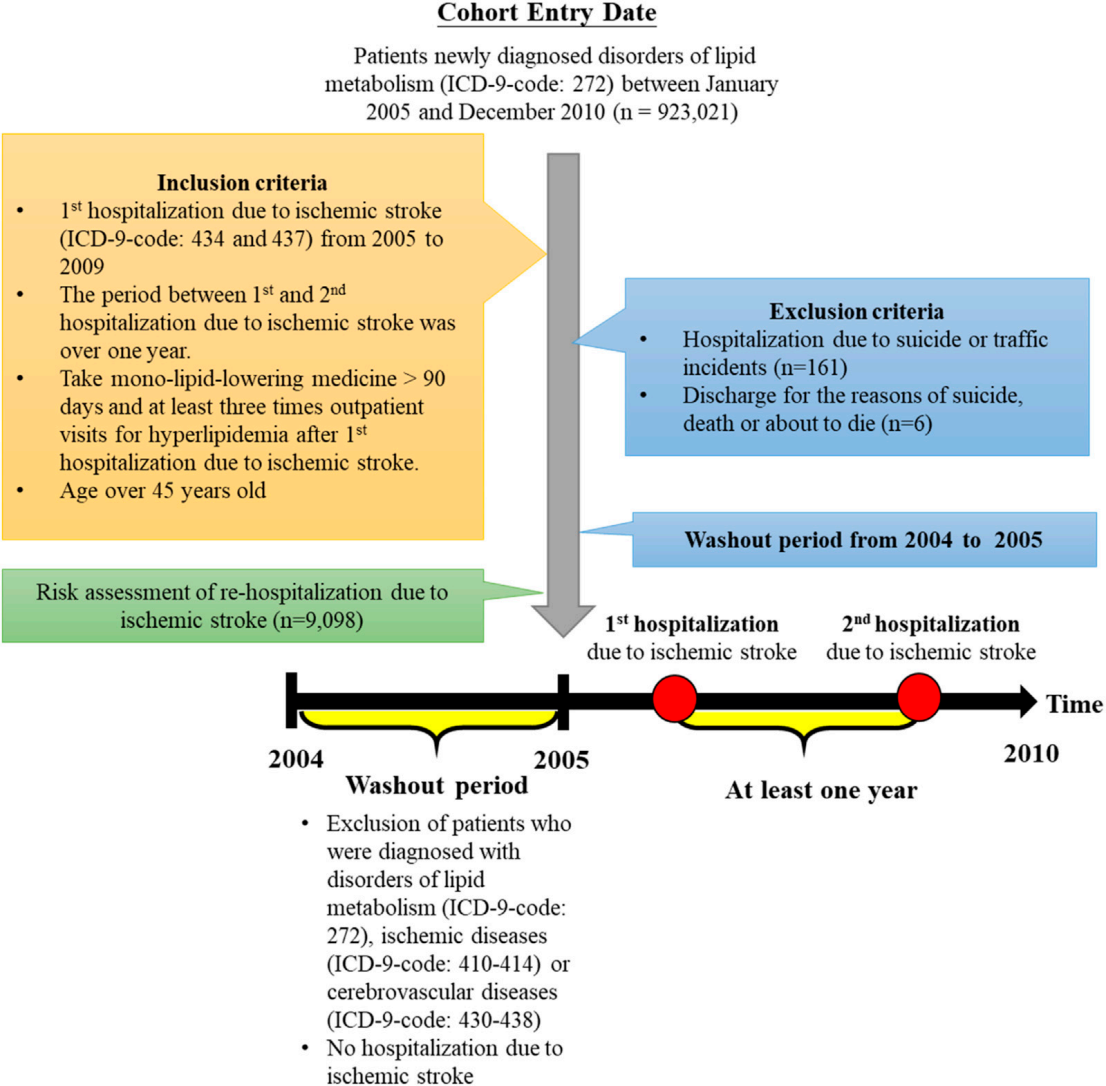
Study workflow.

The coverage rate of National Health Insurance is nearly 100% in Taiwan. The analysis data is from the National Health Insurance Research Database (NHIRD) and capable of representing the whole nation. This is a cohort study and we included the medical claims from 2005 to 2010 in the ICD-9-CM system. During the study period in Taiwan, the clinical description guideline of lipid-lowering medicines was consistent. The eligible criteria of the study population were 1) the new patients with newly diagnosed disorders of lipid metabolism (ICD-9-CM code: 272), 2) the first hospitalization due to IS (ICD-9-CM code: 434 and 437 of inpatient medical records) from 2005 to 2009, 3) taking monolipid-lowering medicine over 90 days and at least three times outpatient visits for hyperlipidemia after the first hospitalization due to IS, 4) age larger than 45 years at which IS likely to occur (Yousufuddin and Young, 2019), 5) the period of time between first and second hospitalization larger than 1 year, and 6) the defined daily dose (DDD) over zero. We excluded the inpatients 1) whose hospitalization cause was car incidents or suicide and 2) who were discharged from the hospitals for the reasons of suicide, death, or about to die. In the end, 9,098 patients are eligible (Figure 1). To diminish the impact of baseline difference of putative confounders, we designed a 1-year washout period prior to the start of the study. Patients who were diagnosed with disorders of lipid metabolism (ICD-9-CM code: 272), ischemic diseases (ICD-9-CM code: 410–414), or cerebrovascular diseases (ICD-9-CM code: 430–438) or who were hospitalized due to IS in 2004 were excluded. Since 2001, the ICD-9-CM system had not been updated (Administration, 2014). Throughout the study period, the diagnosis classification system remained the same. This study was exempted from full review following consultation with the Tri-Service General Hospital Institutional Review Board (TSGH IRB No. B–110–22).

### Blood Lipid-Lowering Medicines

Nine blood lipid-lowering medicines were included (Atorvastatin, Rosuvastatin, Simvastatin, Lovastatin, Pravastatin, Fluvastatin, Bezafibrate, Gemfibrozil, and Fenofibrate). In order to understand the effects of various types of lipid-lowering medicines on the risks of reHospIS, all hyperlipidemic patients were divided into four subgroups according to the type of monolipid-lowering medicine they used on a regular basis: (A) nonstatin lipid-lowering medicines and statins regular users; (B) high-density statins (Atorvastatin, Rosuvastatin, and Simvastatin), nonstatin lipid-lowering medicines (Bezafibrate, Gemfibrozil, and Fenofibrate), and statins regular users; (C) Rosuvastatin, Simvastatin, Lovastatin, Pravastatin, Fluvastatin, Bezafibrate, Gemfibrozil, Fenofibrate, and Atorvastatin regular users; (D) five individual statins (Rosuvastatin, Simvastatin, Lovastatin, Pravastatin, and Fluvastatin) and nonstatin lipid-lowering medicine regular users.

### The Definition of Monolipid-Lowering Medicine Regular Users

Patients who had been prescribed a single type of lipid-lowering medicine for more than 90 days and had at least three times records of outpatient visits from the first hospitalization due to IS to the end of follow-up were classified as monolipid-lowering medicine regular users. In order to clarify the effect of every single type of medication on rehospitalization, patients who used different types of blood lipid-lowering medicines in combination were excluded.

### The Definition of DDD, Compliance Rate, and Comorbidity Diseases

The definition of DDD from WHO is the assumed average maintenance dose per day for a drug used for its main indication in adults. The DDD is a unit of measurement and does not necessarily reflect the recommended or prescribed daily dose (WHO, 2020). The values ranged from 0 to 1. The DDD was calculated between the first hospitalization date due to IS and the end of follow-up.

Compliance rates of lipid-lowering medicines were calculated as the number of days with lipid-lowering medicines supply divided by the total number of days from the first hospitalization date due to IS to the end of follow-up (Wei et al., 2002).

In NHIRD, there is a risk of misclassification bias due to unverified diagnosis coding (Hsieh et al., 2019). As a result, we defined diabetes mellitus (DM) hyperlipidemic patients as those who received medications of comorbidity diseases for over 90 days after the first hospitalization due to IS were defined as having such comorbidity disease. We included the already known comorbidity diseases to IS, i.e., high blood pressure, angina, DM, HF, peripheral arterial occlusion disease, and arrhythmics. Type II diabetes accounted for 99 percent of all diabetes cases in Taiwan. As a result, diabetes was not divided into type I and type II diabetes (National Institutes of He, 2019).

### Statistical Analysis

We used a univariable Cox proportional hazards regression model to explore the association of all indicators, including lipid-blood lowering medicines, characteristics of hospitals, cost of hospitalization, demographic characteristics, and comorbidity diseases with reHospIS. Multiple prediction models of lipid-blood lowering medicines on reHospIS were constructed under the adjustment of significant covariates or confounders by using multivariable Cox proportional hazard model regression. The level of statistical significance was set to be a two-sided *p* value less than 0.05. In the sensitivity analysis, all the hyperlipidemic patients were categorized into four patient subgroups of 1) all hyperlipidemic patients, 2) hyperlipidemic patients with diabetes, 3) nondiabetes hyperlipidemic patients, and 4) nondiabetes and non-HF hyperlipidemic patients. The case number of hyperlipidemic patients with HF was limited (*n* = 99) for further subgrouping and meaningful multivariable statistical analysis (Table 1). Therefore, we did not group study patients by HF. However, we wanted to know the lipid-lowering effects for hyperlipidemic patients without these two comorbidity diseases, and we presented the subgroup of nondiabetes and non-HF hyperlipidemic patients.

**TABLE 1:** Descriptive statistics of study population.

	Rehospitalization due to ischemic stroke (*n* = 9,098)					
	No (*n* = 8,530)		Yes (*n* = 568)		Hazard ratio	*p* values
	*N*	%	*n*	%		
Follow-up time (days, mean, and SD)	1,241	504	935	405		
Year of first-time hospitalization^%^						
2005	1,353	0.16	173	0.30	ref	
2006	1756	0.21	178	0.31	0.97	0.80
2007	1836	0.22	137	0.24	1.02	0.87
2008	1886	0.22	63	0.11	0.77	0.09
2009	1,699	0.20	17	0.03	0.65	0.11
Demographic characteristics						
Age (year, mean, and SD)	64	11	65	10.00	1.01	*
Gender						
Male	4,963	0.58	350	0.62	ref	
Female	3,567	0.42	218	0.38	0.85	0.06
The total cost of first-time hospitalization due to ischemic stroke (United States dollars)^&^						
<667	2,348	0.27	144	0.25	ref	
667–1,000	2,314	0.27	153	0.27	1.12	0.34
1,000–1,333	1,461	0.17	95	0.17	1.12	0.38
≥1,333	2,407	0.28	176	0.31	1.27	*
The total days of first-time hospitalization due to ischemic stroke						
<4	1,216	0.14	73	0.13	ref	
4–7	2,963	0.35	176	0.31	1.00	0.99
7–10	1918	0.22	123	0.22	1.07	0.63
≥10	2,433	0.29	196	0.35	1.33	*
Hospital type						
Public	1855	0.22	137	0.24	ref	
Private	1838	0.22	127	0.22	0.86	0.23
Nonprofit proprietary	4,837	0.57	304	0.54	0.91	0.36
Hospital class						
Medical center	3,546	0.42	205	0.36	ref	
Regional hospital	3,973	0.47	279	0.49	1.21	*
District hospital	1,011	0.12	84	0.15	1.34	*
Location of hospitals						
Taipei capital	2,356	0.28	144	0.25	ref	
Northern	1,144	0.13	85	0.15	1.20	0.18
Central	1,347	0.16	98	0.17	1.19	0.18
Southern	1,467	0.17	99	0.17	1.12	0.38
Southern remote	1877	0.22	121	0.21	1.01	0.95
Eastern	339	0.04	21	0.04	1.03	0.90
Compliance						
DDD						
≤0.1	3,473	0.41	178	0.31	ref	
0.1–0.2	2,523	0.30	182	0.32	1.94	***
0.2–0.3	1248	0.15	84	0.15	2.06	***
0.3–0.4	568	0.07	51	0.09	2.82	***
0.4–0.5	320	0.04	18	0.03	1.95	**
>0.5	398	0.05	55	0.10	4.79	***
Compliance rate						
≤0.25	5,220	0.61	318	0.56	ref	
0.25–0.5	2,551	0.30	156	0.27	1.51	***
>0.5	759	.09	94	0.17	3.16	***
Comorbidities						
High blood pressure (HBP)	6,066	0.71	412	0.73	0.90	0.27
Angina	617	0.07	44	0.08	0.94	0.71
Diabetes mellitus (DM)	2,840	0.33	240	0.42	1.31	**
Heart failure (HF)	87	0.01	12	0.02	1.85	*
Peripheral arterial occlusion disease (PAOD)	604	0.07	46	0.08	1.04	0.80
Arrhythmics	78	0.01	8	0.01	1.52	0.24

***p value < 0.001, **p value < 0.01, *p value < 0.05. Statistical analysis was using univariable Cox proportional hazards regression. ref: reference group and The cost was converted from NT dollars to Unites States dollars at a 30 to one exchange rate. % The period between first hospitalization and second hospitalization due to ischemic stroke was set to be over 1 year, thus no first hospitalization due to ischemic stroke was present in 2010.

## Results

### The Risk Factors of reHospIS

Older age, male sex, higher total cost of first-time hospitalization, higher total days of first-time hospitalization, lower hospital class, developing diabetes and heart failure (HF), and higher DDD and compliance rate were the risk factors of reHospIS (Table 1). DDD and compliance rates tended to indicate the severity of IS in the dose-response effect. Perhaps this is why DDD and compliance rates are positively associated with reHospIS risks.

### The Risks of reHospIS for Hyperlipidemic Patients Taking Monolipid-Lowering Medicine

Hyperlipidemic patients were grouped into four subgroups by the type of monolipid-lowering medicine they took regularly. Four medicine categories were (A) nonstatin lipid-lowering medicines versus statins (served as the reference group in the model, abbreviated as ref), (B) high-density statins (Atorvastatin, Rosuvastatin, and Simvastatin) and nonstatin lipid-lowering medicines (Bezafibrate, Gemfibrozil, and Fenofibrate) versus statins (ref), (C) Rosuvastatin, Simvastatin, Lovastatin, Pravastatin, Fluvastatin, Bezafibrate, Gemfibrozil, and Fenofibrate versus Atorvastatin (ref), and (D) five individual statins of Rosuvastatin, Simvastatin, Lovastatin, Pravastatin, and Fluvastatin versus nonstatin lipid-lowering medicines (ref).

**FIGURE 2:**
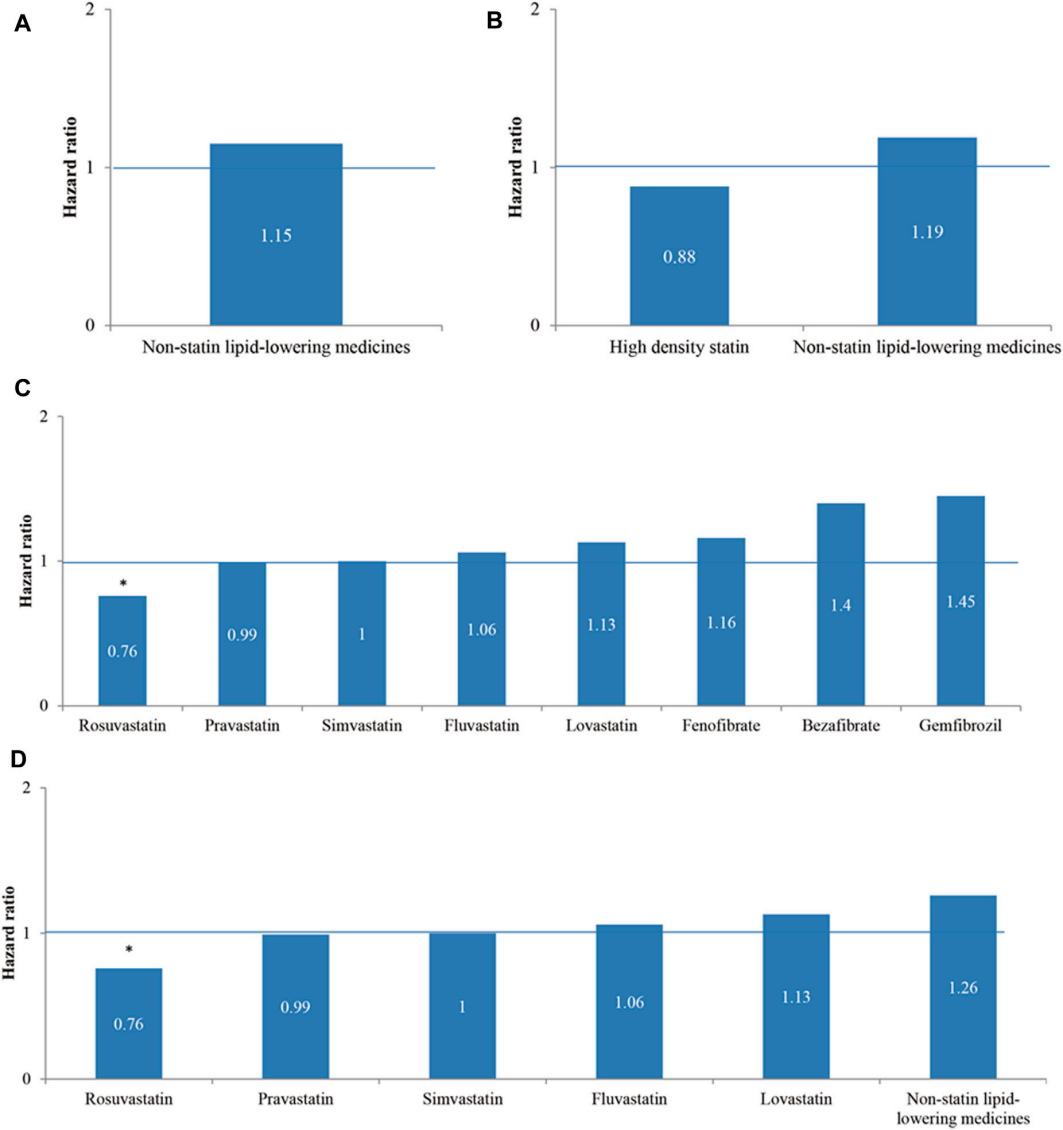
The hazard ratios of rehospitalization due to ischemic stroke (reHospIS) for hyperlipidemic patients grouped by categories of lipid-lowering medicines they took regularly using univariable Cox proportional hazards regression. **(A)** Nonstatin lipid-lowering medicines versus statins (reference group, ref); **(B)** high-density statins (Atorvastatin, Rosuvastatin, and Simvastatin) and nonstatin lipid-lowering medicines versus low-density statins (Bezafibrate, Gemfibrozil, and Fenofibrate) (ref); **(C)** Rosuvastatin, Simvastatin, Lovastatin, Pravastatin, Fluvastatin, Bezafibrate, Gemfibrozil, and Fenofibrate versus Atorvastatin (ref); **(D)** five individual statins of Rosuvastatin, Simvastatin, Lovastatin, Pravastatin, and Fluvastatin versus nonstatin lipid-lowering medicines (ref). The asterisk represents a statistically significant hazard ratio (*p* < 0.05).

In the univariable Cox proportional hazards regression models of four medicine categories (Figure 2), Rosuvastatin regular users had a significantly lower risk of reHospIS (HR = 0.76, *p* < 0.05) than Atorvastatin in subgroups (C) and (D) (Figure 2). Among subgroups (A) to (D), the other lipid-lowering medicines had no significant difference in the risks of reHospIS among each other (Figure 2).

### Multivariable Cox Proportional Hazards Regression Model

We entered all significant factors from univariable Cox proportional hazards regression models (Table 1) and furtherly analyzed them using multivariable Cox proportional hazard regression models. In the sensitivity analysis of evaluating risks of reHospIS, all hyperlipidemic patients are categorized into four patient subgroups: 1) all hyperlipidemic patients, 2) hyperlipidemic patients with diabetes, 3) hyperlipidemic patients without diabetes, and 4) hyperlipidemic patients without diabetes and without HF.

**FIGURE 3:**
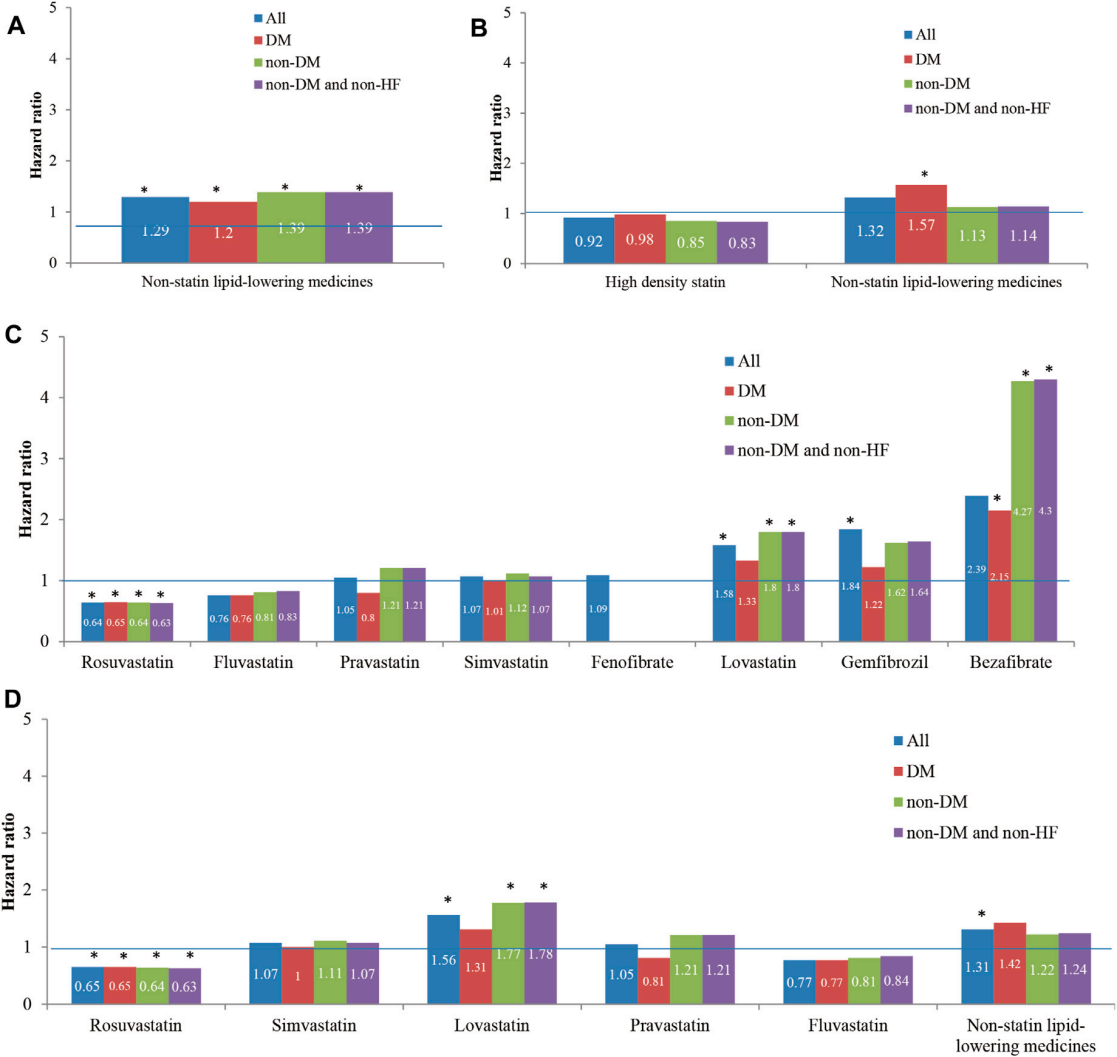
The hazard ratios of reHospIS for hyperlipidemic patients grouped by comorbidities (diabetes mellitus, DM, and heart failure, HF) and categories of lipid-lowering medicines they took regularly using multivariable Cox proportional hazards regression. All the hyperlipidemic patients were categorized into four patient subgroups of 1) all hyperlipidemic patients, 2) hyperlipidemic patients with DM, 3) non-DM hyperlipidemic patients, and 4) non-DM and non-HF hyperlipidemic patients. Cox proportional hazards regression model of each medicine category was conducted for each patient subgroup. The medicine categories were described as follows: **(A)** nonstatin lipid-lowering medicines versus statins (reference group, ref); **(B)** high-density statins (Atorvastatin, Rosuvastatin, and Simvastatin) and nonstatin lipid-lowering medicines versus low-density statins (Bezafibrate, Gemfibrozil, and Fenofibrate) (ref); **(C)** Rosuvastatin, Simvastatin, Lovastatin, Pravastatin, Fluvastatin, Bezafibrate, Gemfibrozil, and Fenofibrate versus Atorvastatin (ref); **(D)** five individual statins of Rosuvastatin, Simvastatin, Lovastatin, Pravastatin, and Fluvastatin versus nonstatin lipid-lowering medicines (ref). The asterisk represents a statistically significant hazard ratio (*p* < 0.05). Significant variables in the univariable Cox proportional hazards regression models were selected and entered in the multiple Cox proportional hazards regression model.

**FIGURE 4:**
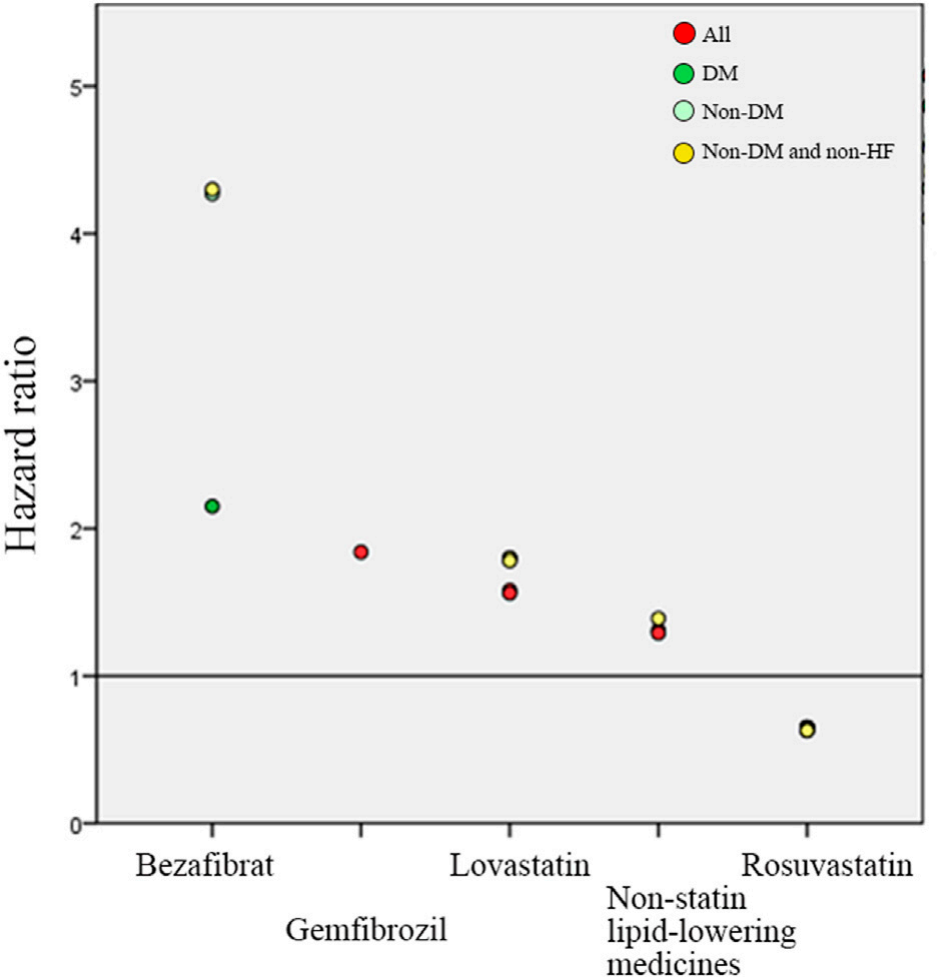
Multivariable Cox hazard ratio regression outcomes of lipid-lowering medicines on predicting the risk of reHospIS. Only significant lipid-lowering medicines are presented. Atorvastatin users were served as the reference group, the horizontal line of value one. There are four hyperlipidemic patient subgroups marked in different colored circles. The red circle is all hyperlipidemic patients (All). The green circle is hyperlipidemic patients with diabetes (DM). The light green circle is non-DM hyperlipidemic patients. The yellow circle is non-DM and none-HF hyperlipidemic patients.

In the results of medicine subgroups (A) to (D), Rosuvastatin regular users had the lowest HRs of reHospIS ranging from 0.63 to 0.64 (*p* < 0.05) for all patients subgroups (Figure 3). Statins regular users had significantly lower risks (HRs = 1.2–1.4) for all patient subgroups as compared with nonstatin lipid-lowering medicines (Figure 3A). Diabetes patients who took nonstatin lipid-lowering medicines had higher risks of reHospIS as compared with those who took low-density statins. There was no statistically significant difference in the risk of reHospIS for all patient subgroups who took low- or high-density statins (Figure 3B). Rosuvastatin regular users of all patient subgroups had the significantly lowest HRs of reHospIS ranging from 0.63 to 0.65 (*p* < 0.05) as compared with Atorvastatin regular users in subgroups (C) and (D) (Figures 3C,D). Lovastatin is one type of statin. Except for the diabetes patient subgroup, the other patient subgroups who took Lovastatin (HR = 1.56–1.78 *p* < 0.05) rather than the other types of statins had the highest risk as compared with Atorvastatin regular users (Figures 3C,D). Except for diabetes patient subgroups, Bezafibrate regular users had significantly higher risks of reHospIS (HR = 2.39–4.3, *p* < 0.05) (Figure 3C). To rule out the possible confounding effect of IS severity, we excluded patients who were not likely to be severe IS patients by excluding low-density statin regular users (the case number of analysis 6,479) (Supplementary Table S1). The abovementioned results remain consistent. Figure 4 depicted the increasing risk trends of reHospIS among regular users of Rosuvastatin, Atorvastatin, nonstatin lipid-lowering medicines, Lovastatin, Gemfibrozil, and Bezafibrate. Except for Bezafibrate, the HR of each blood lipid-lowering medicine in each patient subgroup was similar (Figure 4).

### Bezafibrate and Lovastatin Users With DM Have Lower Risk Than Those Without DM

Though Bezafibrate was linked to a higher risk of reHospIS when compared to Atorvastatin, it appeared to be beneficial to hyperlipidemic patients with diabetes (HR = 2.15, *p* < 0.05) than none DM patients (HR = 4.27, *p* < 0.05) by reducing nearly half the risk of reHospIS. There was no statistical difference of risks of reHospIS in the DM patient subgroup who took Lovastatin (HR = 1.31 *p* = 0.40) or Atorvastatin, whereas Lovastatin was linked to a higher risk of reHospIS for nondiabetes hyperlipidemic patients (HR = 1.8, *p* < 0.05) when compared to Atorvastatin (Figure 3C).

### Summary

Under the adjustments of confounders, statins have a lower risk of reHospIS than nonstatin lipid-lowering medicines users in all subgroups. Among these lipid-lowering medicines, regular Rosuvastatin users had the lowest HRs of reHospIS. Though Bezafibrate and Lovastatin were linked to higher risks of reHospIS, they may be beneficial to DM patients when compared to none DM patients.

## Discussion

This is a 6-years long retrospective study of 9,098 hyperlipidemic patients. Long-term statin users of hyperlipidemic patients had lower reHospIS risks than nonstatin lipid-lowering medicines users. In comparison to Atorvastatin regular users, Rosuvastatin regular users had the lowest HRs of reHospIS among all patient subgroups. Except for hyperlipidemic patients with diabetes, regular Lovastatin users had the highest risks of reHospIS among statins regular users. The increasing trends of risks of reHospIS were observed from Rosuvastatin, nonstatin lipid-lowering medicines, Lovastatin, and Gemfibrozil to Bezafibrate regular users.

The mechanism of statins primarily lowered the concentration of LDL rather than reducing TG or increasing HDL. Randomized trials have shown that lowering LDL cholesterol reduces the risk of stroke (Castilla-Guerra et al., 2019). It may be the reason that statins are more protective against reHospIS than nonstatin lipid-lowering medicines users. The first-generation statins are Pravastatin, Lovastatin, and Fluvastatin; the second-generation statins are Simvastatin and Atorvastatin; and the third-generation statins are Rosuvastatin and Pitavastatin. Second- and third-generation statins were more effective at lowering LDL cholesterol than first-generation statins. In addition, Rosuvastatin outperformed Atorvastatin, Simvastatin, and Pravastatin in terms of LDL-lowering efficacy.

In a mouse experiment, both normal and high doses of Rosuvastatin were found to be effective in preventing rt-PA-associated hemorrhages with brain ischemia while having no effect on cerebral blood reflow or neural function (Lu et al., 2018). In addition, Rosuvastatin slowed the progression of cardiovascular disease in diabetes patients by improving HDL functions and suppressing inflammation. The prevention of unfavorable outcomes of IS was associated not only with LDL-lowering effect but also with pleiotropic effects of endothelial function, modulating thrombogenesis, attenuating inflammatory and oxidative stress damage, and facilitating angiogenesis matters (Zhao et al., 2014).

It was noted that nonstatin lipid-lowering medicines were linked to higher risks of reHospIS in none-diabetes hyperlipidemic patients. Statins are preferred as first-line therapy, and other lipid-lowering medicines should be avoided. However, it has been reported that the combination therapy of statin and nonstatin lipid-lowering medicines (e.g., ezetimibe, fibrates, bile acid sequestrants, PCSK9 inhibitors, and omega-3 fatty acids) (Rodriguez et al., 2018) was recommended for releasing other syndromes. For instance, the combination of Simvastatin and Bezafibrate increased cholesterol efflux in parallel with HDL cholesterol and apoA‐I responses. When compared to statin treatment alone, Bezafibrate and statin combination therapy reduces the risk of 30-day major adverse cardiovascular events and 1-year mortality rates in diabetes patients.

In this study, Bezafibrate regular users with diabetes had nearly 50% lower reHospIS risks than those without diabetes. Bezafibrate is one of the most commonly used molecules in the treatment of hypertriglyceridemia, and statin therapy is often added to achieve lipid profile goals in mixed dyslipidemia (León-Martínez et al., 2021). Bezafibrate ameliorates diabetes and may benefit patients with nonalcoholic fatty liver disease and impaired glucose metabolism by reducing steatosis, enhancing hepatic mitochondrial mass, improving metabolic flexibility, and increasing hepatic insulin sensitivity (Franko et al., 2017).

Lovastatin was an unfavorable lipid-lowering medicine for nondiabetes hyperlipidemic patients due to its high risk of reHospIS, but it had no adverse effects on those with DM when compared to Atorvastatin. Moreover, Lovastatin was found to significantly reduce fatty streak lesion formation in the aortic arch of hyperlipidemic-diabetic hamsters (El-Swefy et al., 2000). Its other functions of lowering plasma total triglycerides and total cholesterols, selectively decreasing non-HDL-C, and providing antioxidant protection may also contribute to its protective effects. The antioxidant effects of Lovastatin may be beneficial for hyperlipidemic patients with diabetes.

There were some limitations in the study. Not all the statins were included in the study (e.g., Pitavastatin) because some statins were not commercially available and proven by Taiwan Food and Drug Administration during the study period. Serum lipid-lowering herbal medicines, oral hypoglycemic agents, lifestyle, and dietary factors were not included. The use of lipid-lowering medicines, blood lipid levels (not available in the NHIRD), and diabetes status of study subjects prior to their first hospitalization due to IS were not included in the study. However, we designed 1-year washout period prior to the start of the study to reduce the impact of the aforementioned conditions.

We only discussed the risk of reHospIS for users of monolipid-lowering medicines. We are unable to assess the combined effects of statins and nonstatin lipid-lowering medicines. However, we discovered that nonstatin lipid-lowering medicines had a beneficial effect on hyperlipidemic patients with diabetes. It calls for further studies into the effects of combinational treatments on the long-term risks of reHospIS.

## Conclusion

In comparison to nonstatin lipid-lowering medicines, statins had a longer-term beneficial effect of secondary prevention of reHospIS for hyperlipidemic patients. Rosuvastatin is the most effective treatment for all subgroups of hyperlipidemic patients. On the other hand, Bezafibrate appears to benefit hyperlipidemic patients with diabetes. The combined effects of statins and nonstatin lipid-lowering medicines on diabetes hyperlipidemic patients warrant further studies to understand the beneficial mechanism.

## Data Availability

The data analyzed in this study is subject to the following licenses/restrictions. The dataset can be applied from the National Health Insurance Research Database according to the application regulation. Requests to access these datasets should be directed to https://nhird.nhri.org.tw/apply_00. html.

## Abbreviations

IS, ischemic stroke; reHospIS, rehospitalization due to IS; HF, heart failure; HBP, high blood pressure; PAOD, peripheral arterial occlusion disease.
