# Supplementary material for: Long-Term Use of Statins Lowering the Risk of Rehospitalization Caused by Ischemic Stroke Among Middle-Aged Hyperlipidemic Patients: A Population-Based Study
Source: Front Pharmacol. 2021 Oct 18;12:741094. doi: 10.3389/fphar.2021.741094 (PMC8558418; doi:10.3389/fphar.2021.741094)
Supplement: Supplementary file 1 [file DataSheet1.docx]

**Long-Term Use of Statins Lowering the Risk of Rehospitalization Caused by Ischemic Stroke Among Middle-Aged Hyperlipidemic Patients: A Population-Based Study**

Jiu-Haw Yin^#^, Dr.^1,2^ , [ch9135@gmail.com](mailto:ch9135@gmail.com)

Giia-Sheun Peng^#^, Dr. ^1,2^, [tsghpeng@gmail.com](mailto:tsghpeng@gmail.com)

Kang-Hua Chen^#^, PhD.^3,4^, khc@mail.cgu.edu.tw

Chi-Ming Chu^#^, Ph.D. ^5,6,7,8,9^, [cm.chu.tw@gmail.com](mailto:cm.chu.tw@gmail.com)

Wu-Chien Chien, Ph.D.^10^, [chienwu@mail.ndmctsgh.edu.tw](mailto:chienwu@mail.ndmctsgh.edu.tw)

Li-Ting Kao, Ph.D. ^5,11,12,13^, [kaoliting@gmail.com](mailto:kaoliting@gmail.com)

Chia-Chao Wu, Dr.^14^, [wucc@mail.ndmctsgh.edu.tw](mailto:wucc@mail.ndmctsgh.edu.tw)

Chih-Wei Yang, Dr.^15^, [youngwayleon@gmail.com](mailto:youngwayleon@gmail.com)

Wen-Chiuan Tsai, Dr.^16^, [ab95057@hotmail.com](mailto:ab95057@hotmail.com)

Wei-Zhi Lin, MS^13,17^ [wz.lin@gapps.ndmctsgh.edu.tw](mailto:wz.lin@gapps.ndmctsgh.edu.tw)

Yi-Syuan Wu, Ph.D.^13^, [pu1254@gmail.com](mailto:pu1254@gmail.com)

Hung-Che Lin, Dr. ^18,19,20^, [lhj50702@gmail.com](mailto:lhj50702@gmail.com)

Yu-Tien Chang*, Ph.D. ^5^ [greengarden720925@gmail.com](mailto:greengarden720925@gmail.com)

**Authors’ affiliations:**

^1^Department of Neurology, Tri-Service General Hospital, National Defense Medical Center, Taipei, Taiwan

^2^Division of Neurology, Department of Internal Medicine, Taipei Veterans General Hospital, Hsinchu Branch, Hsinchu County, Taiwan

^3^Associate Professor, School of Nursing, College of Medicine, Chang Gung University, Tao-Yuan city, Taiwan

^4^Associate Research Fellow, Department of Nursing, Chang Gung Memorial Hospital, Tao-Yuan Branch, Tao-Yuan city, Taiwan

^5^School of Public Health, National Defense Medical Center, Taipei City, Taiwan

^6^Department of Surgery, Songshan Branch of Tri-Service General Hospital, National Defense Medical Center, Taipei City, Taiwan

^7^ Division of Biostatistics and Informatics, Department of Epidemiology, School of Public Health, National Defense Medical Center, Taipei, Taiwan

^8^Department of Public Health, China Medical University, Taichung City, Taiwan

^9^ Department of Healthcare Administration and Medical Informatics College of Health Sciences, Kaohsiung Medical University, Kaohsiung City, Taiwan

^10^Department of Medical Research, Tri-Service General Hospital, National Defense Medical Center, Taipei, Taiwan

^11^Department of Pharmacy Practice, Tri-Service General Hospital, Taipei, Taiwan.

^12^School of Pharmacy, National Defense Medical Center, Taipei, Taiwan.

^13^Graduate Institute of Life Sciences, National Defense Medical Center, Taipei, Taiwan

^14^ Division of Nephrology, Department of Medicine, Tri-Service General Hospital, National Defense Medical Center, Taipei, Taiwan

^15^Division of Gastroenterology, Department of Internal Medicine, Tri-Service General Hospital, National Defense Medical Center, Taipei, Taiwan

^16^Department of Pathology, Tri-Service General Hospital, National Defense Medical Center, Taipei, Taiwan

^17^ School of Medicine, National Defense Medical Center, Taipei City, Taiwan

^18^Graduate Institute of Medical Sciences, National Defense Medical Center, Taipei, Taiwan

^19^Department of Otolaryngology-Head and Neck Surgery, Tri-Service General Hospital, National Defense Medical Center, Taipei, Taiwan

^20^Hualien Armed Forces General Hospital, Hualien County, Taiwan

# Equal contribution.

***Corresponding authors:**

PhD. Yu-Tien Chang

Tel.: +886-2-87923100 ext. 18014

Address: No.161, Sec. 6, Minquan E. Rd., Neihu Dist., Taipei City 11490, Taiwan (R.O.C.)

Email: [greengarden720925@gmail.com](mailto:greengarden720925@gmail.com)

## Table S 1 Multivariable Cox-Hazards models of reHospIS for hyperlipidemic patients excluding low density statin users.

|  | Rehospitalization due to IS | | | | Model 1^$^ | | Model 2 | | Model 3 | |
| --- | --- | --- | --- | --- | --- | --- | --- | --- | --- | --- |
|  | No | | Yes | | HR | p | HR | p | HR | p |
|  | n/mn | %/sd | n/mn | %/sd |  |  |  |  |  |  |
| **Lipid-lowering drug groups** | | | | | | | | | | |
| **Group 1 of individual high density Statin** | | | | | | | | | | |
| Atorvastatin | 3276 | 0.94 | 219 | 0.06 |  | ref. |  | ref. | - | - |
| Rosuvastatin | 1800 | 0.96 | 79 | 0.04 | 0.76 | * | 0.65 | ** | - | - |
| Simvastatin | 1035 | 0.94 | 70 | 0.06 | 1 | 0.991 | 1.06 | 0.674 | - | - |
| **Group 2 of Statins and other lipid-lowering medicines** | | | | | | | | | | |
| Statins | 1800 | 0.96 | 79 | 0.04 | - | ref. | - | - | - | ref. |
| Non-statin lipid-lowering medicines | 4311 | 0.94 | 289 | 0.06 | 1.31 | * | - | - | 1.56 | ** |
| **Compliance** | | | | | | | | | | |
| **DDD rate** |  |  |  |  |  |  |  |  |  |  |
| ≤0.1 | 2463 | 0.96 | 112 | 0.04 |  | ref. |  | ref. |  | ref. |
| 0.1~0.2 | 1869 | 0.94 | 123 | 0.06 | 2 | *** | 2.13 | *** | 2.13 | *** |
| 0.2~0.3 | 928 | 0.94 | 56 | 0.06 | 2.08 | *** | 2.09 | *** | 2.09 | *** |
| 0.3~0.4 | 394 | 0.92 | 35 | 0.08 | 3.09 | *** | 2.88 | *** | 2.89 | *** |
| 0.4~0.5 | 210 | 0.95 | 11 | 0.05 | 1.97 | * | 2.03 | * | 2.03 | * |
| >0.5 | 247 | 0.89 | 31 | 0.11 | 4.94 | *** | 3.91 | *** | 3.92 | *** |
| **Compliance rate** |  |  |  |  |  |  |  |  |  |  |
| ≤0.25 | 3561 | 0.95 | 191 | 0.05 |  | ref |  | ref |  | ref |
| 0.25~0.5 | 1937 | 0.95 | 105 | 0.05 | 1.49 | ** | 0.99 | 0.956 | 0.99 | 0.93 |
| >0.5 | 613 | 0.89 | 72 | 0.11 | 3.28 | *** | 1.76 | ** | 1.75 | ** |
| **Comorbidity** | | | | | | | | | | |
| **High Blood Pressure (HBP)** | 4384 | 0.94 | 264 | 0.06 | 0.82 | 0.09 | - | - | - | - |
| **Angina** | 455 | 0.94 | 31 | 0.06 | 1 | 0.99 | - | - | - | - |
| **Diabetes mellitus (DM)** | 1962 | 0.93 | 152 | 0.07 | 1.31 | * | 1.4 | ** | 1.4 | ** |
| **Heart failure (HF)** | 63 | 0.89 | 8 | 0.11 | 1.87 | 0.08 | - | - | - | - |
| **Peripheral arterial occlusion disease (PAOD)** | 406 | 0.94 | 26 | 0.06 | 0.97 | 0.88 | - | - | - | - |
| **Arrhythmics** | 56 | 0.93 | 4 | 0.07 | 1.21 | 0.70 | - | - | - | - |
| **Demographic characteristics** | | | | | | | | | | |
| Age | 65 | 10 | 66 | 10 | 1.01 | 0.04 | 1.01 | ** | 1.01 | ** |
| Female | 2635 | 0.95 | 142 | 0.05 | 0.82 | 0.07 | 0.74 | ** | 0.74 | ** |
| **Characteristics of hospitalization due to IS** | | | | | | | | | | |
| **The total cost of first-time hospitalization due to IS** | | | | | | | | | | |
| <20,000 | 1640 | 0.95 | 90 | 0.05 |  | ref. |  | ref. |  | ref. |
| 20,000~30,000 | 1610 | 0.95 | 91 | 0.05 | 1.08 | 0.61 | 1.13 | 0.47 | 1.13 | 0.47 |
| 30,000~40,000 | 1085 | 0.95 | 58 | 0.05 | 1.03 | 0.86 | 1.05 | 0.82 | 1.05 | 0.81 |
| ≥ 40,000 | 1776 | 0.93 | 129 | 0.07 | 1.39 | * | 1.25 | 0.33 | 1.25 | 0.33 |
| **The total days of first-time hospitalization due to IS** | | | | | | | | | | |
| <4 | 829 | 0.95 | 42 | 0.05 |  | ref. |  | ref. |  | ref. |
| 4~7 | 2115 | 0.95 | 112 | 0.05 | 1.04 | 0.83 | 1.04 | 0.85 | 1.04 | 0.86 |
| 7~10 | 1383 | 0.95 | 77 | 0.05 | 1.1 | 0.61 | 1.03 | 0.88 | 1.03 | 0.90 |
| ≥ 10 | 1784 | 0.93 | 137 | 0.07 | 1.48 | * | 1.31 | 0.29 | 1.3 | 0.30 |
| **Hospital type** | | | | | | | | | | |
| Public | 1215 | 0.94 | 73 | 0.06 |  | ref. |  |  |  |  |
| Private | 1351 | 0.94 | 87 | 0.06 | 0.95 | 0.76 |  |  |  |  |
| Non-Profit Proprietary | 3545 | 0.94 | 208 | 0.06 | 1.02 | 0.86 |  |  |  |  |
| **Hospital class** | | | | | | | | | | |
| Medical center | 2638 | 0.95 | 141 | 0.05 |  | ref. | - | ref. | - | ref. |
| Regional hospital | 2770 | 0.94 | 170 | 0.06 | 1.17 | 0.17 | 1.36 | ** | 1.36 | ** |
| District hospital | 703 | 0.93 | 57 | 0.08 | 1.43 | * | 1.99 | *** | 2.01 | *** |
| **The region of hospital** | | | | | | | | | | |
| Capital (Taipei) | 1655 | 0.95 | 95 | 0.05 |  | ref. |  |  |  |  |
| Northern | 857 | 0.94 | 59 | 0.06 | 1.18 | 0.33 |  |  |  |  |
| Central | 944 | 0.94 | 63 | 0.06 | 1.15 | 0.39 |  |  |  |  |
| Southern | 1022 | 0.95 | 59 | 0.05 | 1.03 | 0.85 |  |  |  |  |
| Southern remote | 1379 | 0.95 | 80 | 0.05 | 0.92 | 0.57 |  |  |  |  |
| Eastern | 254 | 0.95 | 12 | 0.05 | 0.85 | 0.60 |  |  |  |  |

^$^Model 1 was the results of univariable Cox proportional hazards regression model.

Model 2-3 were the results of multiple variable Cox proportional hazards regression models.

ref: reference group

## Table S 2 The prescribed medications for comorbidity diseases

| **Drug type** | **Angina** | **Arrhythmics** | **Diabetes** | **High Blood Pressure (HBP)** | **Heart failure (HF)** | **Peripheral arterial occlusion disease (PAOD)** | **The number of drug products** |
| --- | --- | --- | --- | --- | --- | --- | --- |
| Acei |  |  |  | 126 |  |  | 126 |
| Anti-anginals | 114 |  |  |  |  |  | 114 |
| Anti-arrhythmics |  | 19 |  |  |  |  | 19 |
| Arb |  |  |  | 27 |  |  | 27 |
| Beta blockers |  |  |  | 240 |  |  | 240 |
| Calcium blockers |  |  |  | 148 |  |  | 148 |
| Digoxin |  |  |  |  | 19 |  | 19 |
| Insulin |  |  | 88 |  |  |  | 88 |
| Loop diuretics |  |  |  | 91 |  |  | 91 |
| Oral anti-dm drug |  |  | 367 |  |  |  | 367 |
| Platelet aggregation inhibitors (antiplatelet) |  |  |  |  |  |  | 171 |
| Potassium-sparing diuretic |  |  |  | 46 |  |  | 46 |
| Pvd drugs |  |  |  |  |  | 109 | 109 |
| Thiazide diuretics |  |  |  | 102 |  |  | 102 |
| Vitamin k antagonists |  |  |  |  |  |  | 10 |
| Sum | 114 | 19 | 455 | 780 | 19 | 109 | 1677 |
